# Ambler’s Journal of Dental Operations

**Published:** 1849-04

**Authors:** 


### AMBLER'S JOURNAL OF DENTAL OPERATIONS.

THIS work, so valuable to every dentist, arranged to suit individual accounts, with blank pages on which to note important items in connection with every case, we are glad to see is still supplied in different size and binding, to suit the profession. We are satisfied the profession would be much benefitted were they more generally kept, and all important facts recorded. In this way every practitioner could soon collect such a number of items professional, that it would be no trouble to supply our dental periodicals with the most valuable information.

Dr. Brown, Dental Depot, Cincinnati, keeps the work for sale.—[CIN. ED.

